# Microplasma direct writing for site-selective surface functionalization of carbon microelectrodes

**DOI:** 10.1038/s41378-019-0103-0

**Published:** 2019-11-18

**Authors:** Aung Thiha, Fatimah Ibrahim, Shalini Muniandy, Marc J. Madou

**Affiliations:** 1 Department of Biomedical Engineering, Faculty of Engineering, University of Malaya, 50603 Kuala Lumpur, Malaysia; 2 Centre for Innovation in Medical Engineering (CIME), Department of Biomedical Engineering, Faculty of Engineering, University of Malaya, 50603 Kuala Lumpur, Malaysia; 3 Nanotechnology and Catalysis Research Centre, Institute of Graduate Studies, University of Malaya, 50603 Kuala Lumpur, Malaysia; 4 Department of Biomedical Engineering, University of California, Irvine, CA 92697 USA; 5 Department of Mechanical and Aerospace Engineering, University of California, Irvine, CA 92697 USA

**Keywords:** Structural properties, Chemistry, Electrical and electronic engineering, Biosensors

## Abstract

Carbon micro- and nanoelectrodes fabricated by carbon microelectromechanical systems (carbon MEMS) are increasingly used in various biosensors and supercapacitor applications. Surface modification of as-produced carbon electrodes with oxygen functional groups is sometimes necessary for biofunctionalization or to improve electrochemical properties. However, conventional surface treatment methods have a limited ability for selective targeting of parts of a surface area for surface modification without using complex photoresist masks. Here, we report microplasma direct writing as a simple, low-cost, and low-power technique for site-selective plasma patterning of carbon MEMS electrodes with oxygen functionalities. In microplasma direct writing, a high-voltage source generates a microplasma discharge between a microelectrode tip and a target surface held at atmospheric pressure. In our setup, water vapor acts as an ionic precursor for the carboxylation and hydroxylation of carbon surface atoms. Plasma direct writing increases the oxygen content of an SU-8-derived pyrolytic carbon surface from ~3 to 27% while reducing the carbon-to-oxygen ratio from 35 to 2.75. Specifically, a microplasma treatment increases the number of carbonyl, carboxylic, and hydroxyl functional groups with the largest increase observed for carboxylic functionalities. Furthermore, water microplasma direct writing improves the hydrophilicity and the electrochemical performance of carbon electrodes with a contact-angle change from ~90° to ~20°, a reduction in the anodic peak to cathodic peak separation from 0.5 V to 0.17 V, and a 5-fold increase in specific capacitance from 8.82 mF∙cm^−2^ to 46.64 mF∙cm^−2^. The plasma direct-writing technology provides an efficient and easy-to-implement method for the selective surface functionalization of carbon MEMS electrodes for electrochemical and biosensor applications.

## Molecular modifications, made to order

A new approach for controlled ‘writing’ with a microscale plasma stream enables precise and efficient chemical modification of three-dimensional carbon electrodes. Such materials are highly desirable for diverse microelectronics applications, but conventional plasma-based techniques for targeted modification are relatively labor-intensive. Fatimah Ibrahim and colleagues at the University of Malaya have devised a novel strategy in which a controllable conductive microelectrode is used to precisely direct a microplasma discharge at specific sites on the surface of a carbon electrode. As a demonstration, they introduce water vapor into the system, thereby coupling of oxygen molecules to the electrodes. This approach enabled them to increase the amount of surface oxygen by nearly ten-fold, enabling efficient subsequent modification with other chemical groups, and the authors demonstrate that the resulting electrodes could offer superior performance for biosensors and other applications.

## Introduction

Carbon microelectromechanical system (carbon MEMS) techniques enable micro- and nanofabrication of three-dimensional carbon electrodes^1^. In a typical carbon MEMS process, patterned polymer photoresists are pyrolyzed to obtain carbon microstructures. The potential of the pyrolytic microelectrodes in electrochemical electrodes for biosensing and energy-storage applications has been well established^2–6^. As with other carbon-based electrodes, such as those based on carbon nanotubes or graphene, surface modification of the electrode surface is often necessary to activate the carbon MEMS electrode surface. For biofunctionalization^2,3,7^ and better electrochemical performance^6,8^, oxygen functional groups must be introduced. The possible oxygen-containing functional groups on a carbon surface include epoxyl, carboxyl, carbonyl, phenol, quinone, and lactone groups in various C=O, O–C=O, and C–OH bond arrangements. Electrochemical treatment, strong acid treatment, vacuum UV treatment, and oxygen plasma treatment are currently the most established techniques to increase oxygen functionalities on a pyrolyzed carbon surface^3,7,9^. Hirabayashi et al. investigated different techniques for carboxyl group functionalization of pyrolyzed carbon and found that oxygen plasma surface treatment is more reliable and less damaging to a carbon electrode while producing more oxygen functional groups in significantly less time than acid treatment^3^.

Oxygen plasma treatment of carbon creates hydrophilic carboxyl and hydroxyl bonds with improved wettability^10^ and surface potential^11^. The carboxylation of carbon electrodes plays an essential role in biosensor fabrication because carboxylic groups can be cross-linked with terminal amines from biomolecules through carbodiimide cross-linker chemistry^2,9^. Moreover, hydroxyl and carboxyl groups on carbon electrodes have been found to significantly improve the specific capacitance and electron transfer rate for supercapacitors and energy-storage applications^12–16^. In a typical conventional plasma treatment, a low-pressure plasma is created by a radio-frequency power source in a vacuum chamber. Ions from the plasma react with the target placed on the cathode in a low-pressure chamber, resulting in the desired surface-functional groups. This plasma setup comprises a low-pressure environment in a large and high-cost setup. In addition, a conventional plasma treatment is not able to target specific spots on a surface without using photoresist-masking techniques that require more complex photolithography and lift-off steps. Hence, atmospheric-pressure microscale plasma (microplasma) jets are increasingly used for localized chemical treatment and targeted nanomaterial synthesis in a wide range of applications^17–19^. Unlike in a conventional plasma treatment, a microplasma jet setup is lightweight, low cost, and low power and operates under atmospheric conditions^17^.

The most common microplasma jets described in the literature feature high-voltage electrodes in a nozzle to generate a plasma from a feed gas. A plasma jet is then emitted from a microsized opening to a target a few millimeters downstream^17,20,21^. The feed gas is generally noble gases mixed with a desired precursor gas (e.g., a mixture of argon and oxygen). In contrast, the plasma direct-writing setup introduced in this paper uses a direct microplasma discharge created between a conductive microelectrode tip in the nozzle and the conductive surface of a target material. Hence, this configuration eliminates both the need for a microscale orifice and noble gases. This type of direct writing with a plasma has been used, for example, to reduce metallic ions on a surface^22^. The physics behind the plasma ignition and its propagation from the needle to the plane surface is well established^23–25^. When the electric field at the sharp tip of the needle electrode is large enough, air near the tip becomes ionized and conductive. As the electric field accelerates the ions and free electrons, they collide with gas molecules in their path, multiplying ions and electrons in a process known as the Townsend avalanche. The resulting corona discharge propagates from the needle tip to the plane electrode. Under high-current conditions, the corona streamer discharge can evolve into an electric arc.

By integrating the plasma needle electrode in a 3-axis motion platform, an automated plasma direct-writing system can be constructed. In this work, we used plasma direct writing to pattern oxygen-functional groups on carbon MEMS electrode surfaces by feeding water vapor into the plasma discharge. It has been shown that a water plasma contains various active species such as H_2_O^+^, OH^+^, H^+^, OH^−^, O^−^, and H^−^, which can be useful for the carboxylation of carbon electrode surfaces^26,27^. We evaluated the oxidation degree of SU-8-derived pyrolyzed carbon surfaces in terms of their carbon-to-oxygen (C/O) ratios. The plasma-written patterns of oxygen-functional groups were observed with energy-dispersive X-ray spectroscopy (EDS) elemental mapping and X-ray photoelectron spectroscopy (XPS). We also explored the effects of surface-functional groups on the electrochemical characteristics for the purpose of biosensing applications. Functionalization of carbon electrodes with oxygen functionalities is an essential step toward their applications in electrochemical sensors^4,6^ and biosensors^2,3^. The ability to pattern oxygen-functional groups is important for mask-less site-selective surface functionalization of carbon microelectrodes for sensor fabrication. In combination with advances in printed manufacturing of carbon materials^28^, this functional group patterning has potential applications in remote and distributed manufacturing of chemical and biological sensors.

## Results and discussion

### Plasma direct-writing setup

**Fig. 1:**
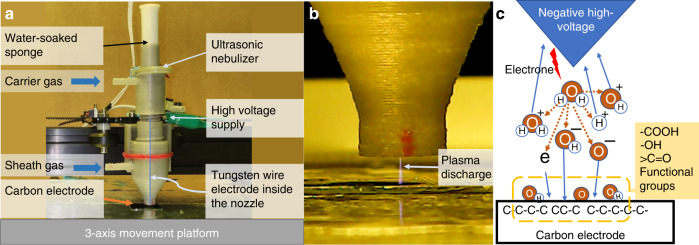
Plasma direct-writing system experimental setup. **a** Plasma direct-writing nozzle system. **b** Plasma streamer (filamentary discharge) from the nozzle to a carbon electrode on a silicon wafer. **c** Schematic representation of the water molecule ionization mechanism and surface functionalization of the carbon electrode

As shown in Fig. 1, our homebuilt experimental plasma direct-writing setup consists of a filamentary plasma discharge (plasma streamer) generated from a tungsten microelectrode tip, a water vapor supply to the plasma streamer, and a 3-axis motion control platform. The operational details of the setup are described in the “Materials and methods” section. A high-voltage source ignites the microplasma streamer discharge between a tungsten microelectrode tip (100-μm diameter) and a carbon electrode positioned on a conductive platform. The carbon electrode is a 10-μm-thick microfilm fabricated from patterned SU-8 on silicon wafer through the carbon MEMS process. The plasma discharge ionizes water molecules to H_2_O^+^, OH^+^, H^+^, OH^−^, O^−^, and H^−^ ions^26,27^. The plasma discharge can be either a positive or negative streamer depending on the biasing of the voltage source to the tungsten microelectrode. The resulting ions bombard the carbon electrode surface, breaking carbon-to-carbon bonds and creating C–OH and C–O–OH bonds in the process.

**Fig. 2:**
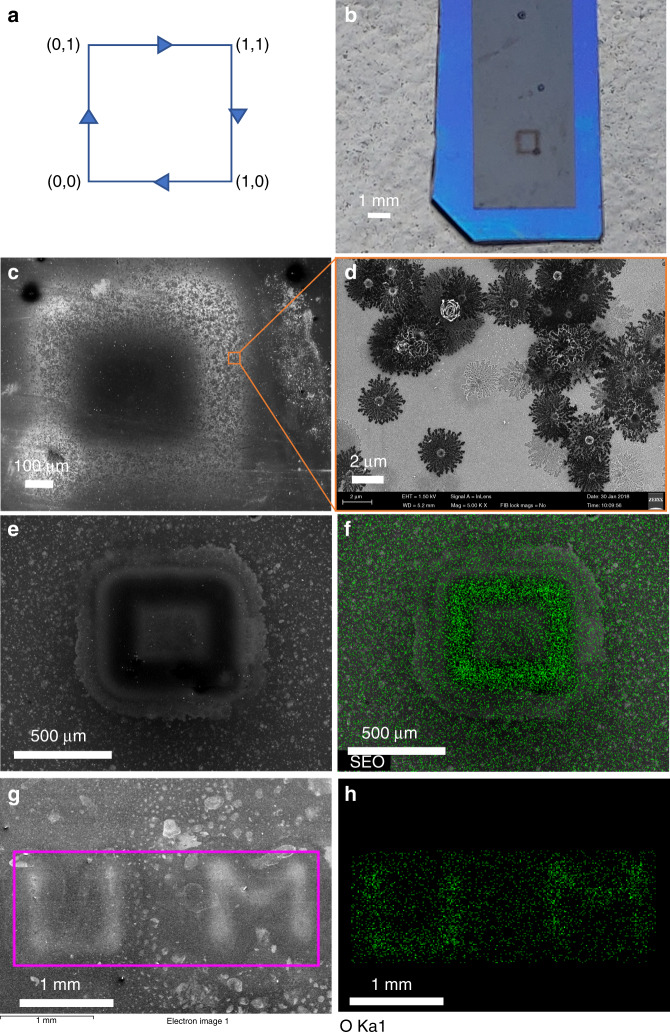
FESEM and EDS micrographs of direct-writing patterns. **a** Schematic diagram showing the programmed tip movement direction of plasma direct writing of a 1-mm square pattern. **b** Optical image of the carbon film electrode showing plasma-treated patterns. **c** FESEM image showing direct writing of a 500-μm square pattern with a positive plasma streamer. **d** Magnification of a treated area from **c** showing flower-like patterns. **e** An FESEM image showing plasma direct writing of a 500-μm square with a negative plasma streamer. **f** EDS elemental mapping of oxygen superimposed on an image (**e**). **g** An arbitrary pattern “UM” drawn with negative plasma direct writing. **h** EDS elemental mapping of oxygen from (**g**)

Figure 2a shows a schematic of plasma microelectrode movement direction during patterning. An optical image of the pattern on the carbon film electrode is presented in Fig. 2b. From the EDS measurements, positive plasma writing was found to result in a slight increase of oxygen-functional groups (from ~5 to ~6%) on the surface. The EDS analysis also reveals the presence of tungsten (~1%) on the carbon surface, which indicates some decomposition of the tungsten plasma electrode. Tungsten is absent in the negative streamer-treated surface because tungsten ionizes into positive ions. In Fig. 2, we compare the results of direct writing by positive and negative plasma streamers on pyrolyzed carbon films. The positive ion treatment resulted in microdots on the carbon surface (Fig. 2c). At higher magnification, those microdots reveal microflower-like branching patterns (Fig. 2d), also known as Lichtenberg figures^29^. These patterns indicate electrical breakdown of the carbon material. It has been shown experimentally that a water plasma contains a much higher density of positive ions (mostly H_2_O^+^) compared with negative ions (OH^–^)^26,27^. Hence, the positive streamer with its higher ion concentration yields higher currents, causing electrical breakdown. These patterns were also observed with a negative plasma when the plasma progressed into an arc discharge. As the reactivity of the plasma depends on the streamer current density, the high-current positive plasma streams and high-current electric arcs have an etching effect on the carbon electrode, revealing the underlying silicon substrate. By limiting the plasma current with a high-impedance load, the negative corona stream discharge can be stabilized to prevent it from progressing into an arc discharge. By using negative plasma direct writing, we can write fine patterns of oxygen groups, as shown in Fig. 2e–h. The accompanying EDS elemental maps confirm the patterns of oxygen functionalities in the plasma direct-written areas.

### Carbon-to-oxygen ratio

**Fig. 3:**
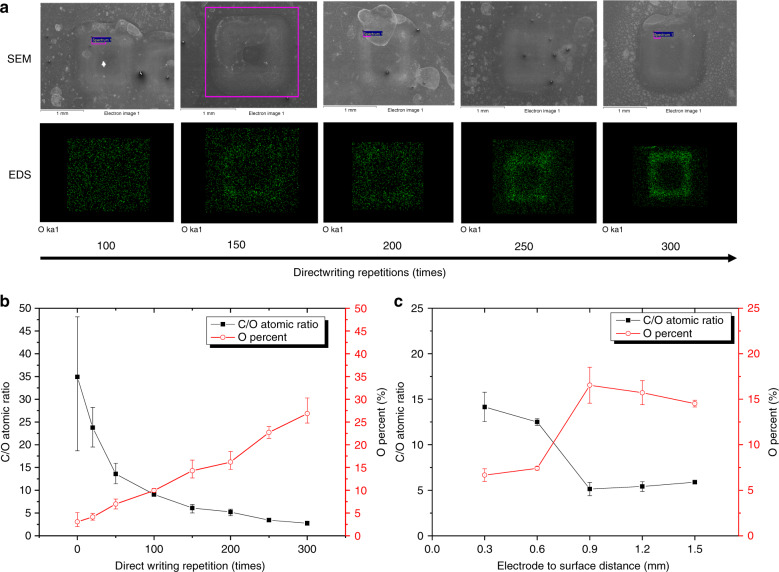
Effect of plasma direct-writing parameters on the oxygen concentration. **a** FESEM and EDS elemental mapping of O over increasing exposure. Atomic percentage of oxygen and atomic C/O ratio over **b** various plasma direct-writing repetitions at a 1-mm tungsten electrode tip to the carbon surface distance and **c** various electrode tips to surface distances at 200 writing repetitions (*N* = 3–6)

Figure 3a shows a series of FESEM and EDS micrographs of 1-mm square plasma direct-written patterns. The programmed tip movement of the patterns is illustrated in Fig. 2a. In microplasma direct writing, the plasma stream passes over a spot many times during a functionalization treatment. Hence, the overall process time does not represent the total exposure time of a treated area, unlike in conventional plasma treatment. Here, we measure the exposure by the number of writing scans during a treatment. In all experiments, the plasma scan rate was 400 mm min^−1^ (6.67 mm s^−1^). For the 1-mm square pattern shown in Fig. 3a, 100 writing scans theoretically equal 60 s of exposure [100 × (4 mm/6.67 mm s^−1^)]. However, as the 3-axis movement control system has a delay of a few milliseconds between the execution of each machine code command, the total time is slightly longer. This delay also explains the uneven functionalization of a pattern resulting in more oxidation at the corners of the square pattern as the nozzle remains stationary there for milliseconds before moving on (see Fig. 3a at 250 repetitions). However, this effect is mitigated for higher exposure times as oxygen groups become saturated.

Figure 3b shows a reduction in the C/O ratio, i.e., an increase in the oxygen concentration, as the number of direct-writing repetitions increases. The C/O ratios are calculated from atomic carbon and oxygen percentages obtained from the EDS spot and area scans. In our experiments, we achieved a minimum C/O atomic ratio of 2.75 ± 0.4 (corresponding to 2.06 ± 0.29 by weight ratio). This result is markedly better than that obtained for surface treatment of SU-8 pyrolyzed carbon by using chamber plasma or acid treatment, as reported previously^3^; the minimum C/O ratios by weight percentage reported were ~4.9 for both plasma and strong acid treatments. Here, we achieved a maximum surface oxidation of 26.85 ± 3% by atom percent after 300 passes. The decrease in the C/O ratio plateaued as the writing repetitions increased further, and the carbon surface became saturated with oxygen-functional groups, leaving little room for further functionalization. This phenomenon has been observed in a previous study on the plasma treatment of carbon nanotubes^30^. For comparison, the C/O ratio achieved in this study approaches that observed for unreduced graphene oxide^31,32^. While graphene oxide is not conductive, an oxygen-functionalized pyrolyzed carbon structure is electrically conductive^3^. Note that the large error bar in the C/O ratio (18–48) for the untreated carbon in Fig. 3b is produced by a minor variation in the amount of the corresponding oxygen percent (5–2%).

We also investigated the effect of the distance between the tungsten electrode tip and the carbon surface on the oxygen concentration of direct-written patterns. The graph in Fig. 3c shows that there is no significant increase in the oxygen concentration at the surface when the distance is less than 0.6 mm. The oxygen concentration markedly improves at a distance of approximately 1 mm, and then, at yet larger distances, is slightly reduced again. With the smallest electrode gaps, the secondary ion emission from the Townsend avalanche is lower because of fewer gas molecules in the discharge path, which may cause a lower degree of functionalization. On the other hand, as the electrode distance becomes greater, the plasma ion energy is reduced, which also results in a lower oxygen concentration.

In terms of writing resolution, EDS elemental mapping was used to measure the line widths of the plasma-written patterns. Oxygen patterns in elemental maps were only observed once the surface oxygen concentration was higher than ~15%. As such, we were unable to measure pattern resolutions acquired under 200 writing scans. For the samples that could be measured, we found that the line width variation is insignificant for various writing repetitions and electrode gaps. The line width of the direct-written patterns averages 141 µm with a standard deviation of 30 µm (*N* = 12). This resolution is in line with microplasma jet printing of nanomaterials previously reported^20^. To test the lifetime of the plasma-written surface functionalization, we examined the oxygen concentration after 3 months of storage of the patterned carbon samples under normal room conditions. EDS scanning of plasma-treated spots showed that the oxygen concentration decreased by an average of 1.9% (*N* = 4), while the untreated areas remained the same.

### XPS analysis

**Fig. 4:**
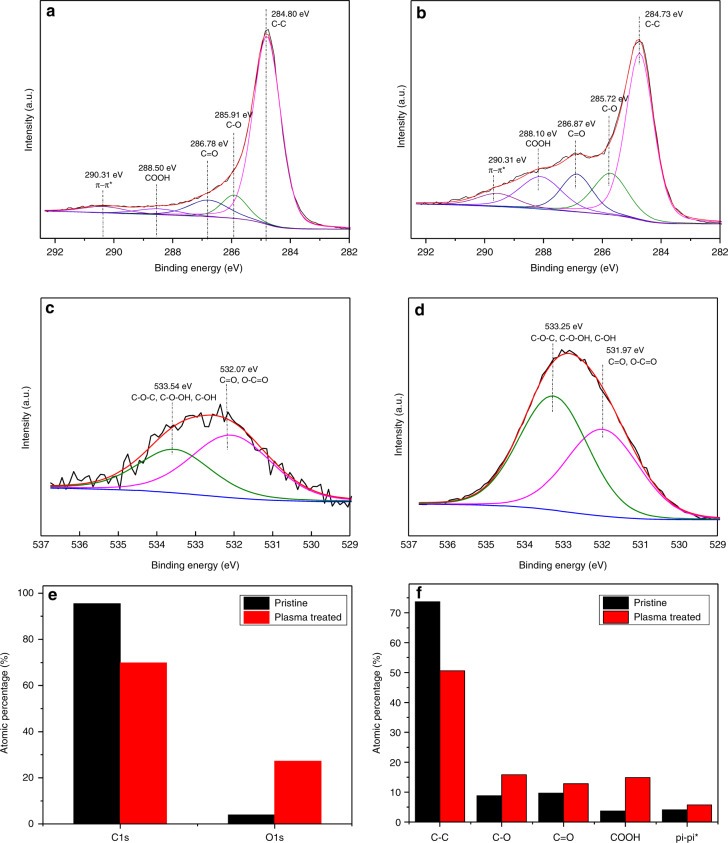
Results of the XPS analysis of carbon electrodes in pristine and treated areas. High-resolution XPS spectra of C1s of **a** pristine and **b** plasma direct-writing samples. High-resolution XPS of O1s of **c** pristine and **d** plasma-treated samples. **e** Atomic percentage of C1s and O1s as measured by XPS. **f** Relative percentage of oxygen-functional groups before and after plasma treatment. Plasma treatment in the graphs was performed at 1-mm distance for 300 repetitions

Although EDS scans can determine the atomic and weight percentages of oxygen and carbon on the electrode surface, it does not reveal the nature of the carbon-to-oxygen bonding such as in carbonyl, hydroxyl, epoxyl, or carboxyl bonding. Hence, XPS is performed to identify the exact nature of the oxygen-containing functional groups on the carbon surface after microplasma direct writing. An area of 3 mm × 2 mm of a carbon film surface was treated by plasma direct writing with the tungsten tip 1 mm from the carbon surface and scanned 300 times. XPS spectra were taken of the pristine and microplasma-treated areas of the same carbon film, and the results are illustrated in Fig. 4. The total atomic oxygen concentration increased from 3.9% in the untreated area to 27.24% in the treated areas (Fig. 4e). Hence, the atomic C/O ratio was reduced from 24.5 to 2.56, which agrees with the EDS measurements discussed above. The C/O ratio in untreated SU-8-derived pyrolyzed carbon was also in agreement with previous literature data, where 3.1% of atomic oxygen was measured by using XPS^33^. In an XPS survey scan of the treated area, there was also a trace amount of sodium and calcium at 1.04% and 1.24%, respectively, indicating possible dissolved salts in the water vapor source.

**Fig. 5:**
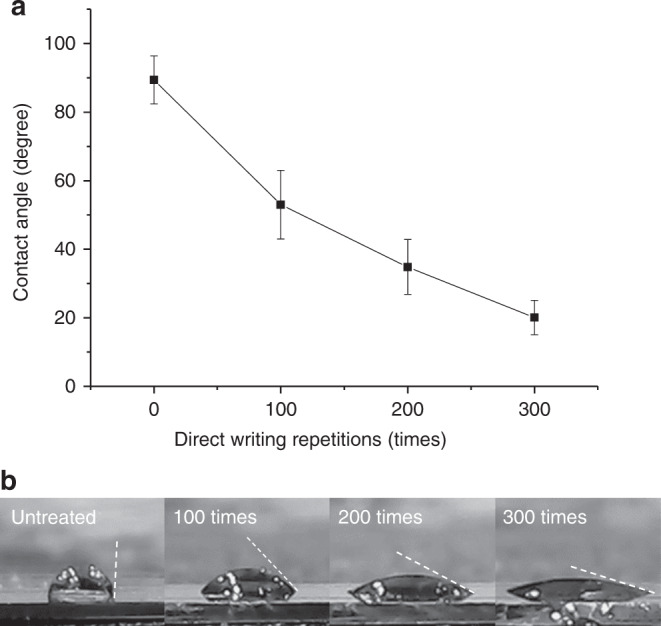
Water droplet contact-angle analysis. **a** Contact angle as a function of plasma direct-writing repetitions (*N* = 3). **b** Representative photographs of water droplets in various direct-writing times

High-resolution XPS spectra were deconvoluted with a nonlinear curve-fitting program. The C1s spectra were deconvoluted into five peaks related to carbon atoms in C–C bonds at 284.80 eV, C–O bonds at 285.91 eV indicating possible phenol, hydroxyl, alcohol, and ether groups, C=O bonds at 286.78 eV indicating carbonyl and quinone groups, and O–C=O groups at 288.50 eV indicating carboxylic groups^34^. The π–π* transitions are evident at 290.31 eV. The results indicate that pristine carbon surfaces feature some oxygen groups that may be remnants of epoxy and phenol groups initially present in the SU-8 precursor. After plasma treatment, the percentage of oxygen functionalities in the C1s spectra increased, and the peaks slightly shifted left. All the carbon-to-oxygen bonds increased after treatment, as evident from Fig. 4f. The most significant change occurs for carboxylic (COOH) groups, which showed a 4-fold enhancement from 3.68% to 14.92% of C1s. The increased carboxylation is important for biomolecule immobilization in the fabrication of carbon MEMS-based biosensors. Deconvolution of the XPS O1s peak resulted in two peaks: one at 533.54 eV and one at 532 eV. The former corresponds to C–O–C and C–OH groups, while the latter indicates C=O groups of carbonyl and carboxylic functional groups^34,35^. Figure 5 shows a reduction in the contact angle with increased plasma-writing repetitions, from ~90° in the untreated surface to ~20° after 300 writing scans. As supported by the XPS analysis, plasma direct writing increases hydrophilic functional groups, such as hydroxyl and carboxyl groups, which have hydrophilic –OH endings. Hence, the wettability of the carbon electrode increases with increasing oxygen- functional groups at longer treatment times.

In Table 1, we present a comparison of various surface treatments on carbon materials and the resultant reduction in the C/O ratio (i.e., increased oxygen percentage). The oxidation degree by surface treatment has been described in various forms in the literature, such as by the C/O ratio^3^, the oxygen-to-carbon (O/C) ratio^36^, and the percentage of oxygen^34,37^, making a direct comparison between different data sources difficult. Here, we converted all values into the C/O ratio for comparison. Among the treatment methods compared, the plasma direct-writing method shows the most significant increase in the oxygen percentage, and it also reduces the treatment time (in small-area treatment).

**Table. 1:** Comparison of the maximum reduction in the C/O ratio achieved with various surface oxidation treatments of carbon electrodes

Surface treatment methods	Carbon materials	Treatment times	Analysis methods	C/O		References	Notes
				Untreated	Treated		
Oxygen plasma treatment	Pyrolyzed carbon	300 s	EDS	19^*^	4.9^*^	^3^	Weight%
Acid treatment (sulfuric, nitric acids)	Pyrolyzed carbon	15 h	EDS	19^*^	4.9^*^	^3^	Weight%
Acid treatment (nitric acid)	Carbon nanotubes	48 h	XPS	24.8	3.44	^34^	C/O calculated from O%
Oxygen plasma treatment	Carbon fiber	9 min	XPS	16.67	5.68	^12^	Converted from the O/C ratio
Electrochemical treatment	Carbon fiber	176.7 min	XPS	6.32	3.8	^36^	Converted from the O/C ratio
Helium plasma touch	Glassy carbon	300 s	XPS	30.25	3.11	^37^	Calculated from C and O%
Plasma direct writing	Pyrolyzed carbon	180 s (300 times)	EDS	25.38 ± 6.87^*^	2.06 ± 0.29^*^	This work	Weight%
			EDS	34.91 ± 10.7	2.75 ± 0.4		Atomic%
			XPS	24.5	2.56		

Unless otherwise noted, C/O ratios are in atomic percent

### Electrochemical characterization

**Fig. 6:**
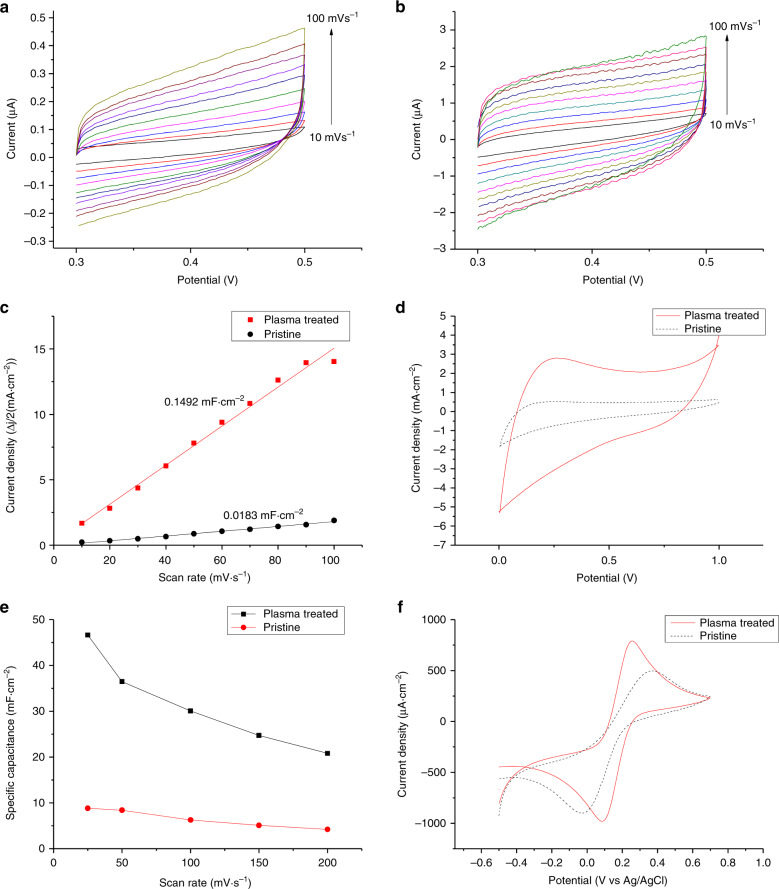
Electrochemical characterization results. Cyclic voltammograms (CV) of **a** pristine carbon and plasma direct-writing carbon **b** surface in 0.5 M H_2_SO_4_ with scan rates from 10 mV s^−1^ to 100 mV s^−1^. **c** Relationship between the scan rate and half of the difference between anodic and cathodic current densities (Δj/2) at 0.4 V. The slope is *C*_dl_. **d** CV of carbon electrodes in 0.5 M H_2_SO_4_ at a 50 mV s^−1^ scan rate. **e** Specific capacitance of carbon electrodes at various scan rates. **f** Electrochemical response of electrodes in 1 mM K_3_ [FeCN_6_]/0.1 M KCL solution at a scan rate of 10 mV s^−1^. (All plasma treatments in the graphs were performed at 1-mm distance for 300 repetitions.)

The effect of surface-bonded oxygen-functional groups on the carbon surface was evaluated by using cyclic voltammetry (CV) in a 0.5 M H_2_SO_4_ solution. Double-layer charging experiments were carried out in a nonfaradaic-charging voltage region, i.e., 0.3–0.5 V at different scan rates from 10 mV s^−1^ to 100 mV s^−1^. The double-layer capacitance (*C*_dl_) was calculated by plotting 1/2 of the difference between the anodic and cathodic current densities (Δj/2) at 0.4 V against the scan rate (*s*). The slope of that plot corresponds to the electrochemical double-layer capacitance (see Fig. 6c). From this plot, the double-layer capacitance of the pristine carbon surface and plasma direct-written carbon surface were found to be 0.0183 mF cm^−2^ and 0.1492 mF cm^−2^, respectively. This increase of ~8 times indicates that microplasma direct writing increases the density of phenolic hydroxyl groups, which improves the double-layer capacitance^14,38^.

CVs of plasma-treated samples by using a three-electrode cell in Fig. 6d reveal Faraday’s current contribution at a broad peak of 0.1–0.4 V, indicating the development of pseudocapacitance. The geometric specific capacitance (*C*_s_) of the carbon electrodes was evaluated in a larger-potential window from 0 to 1 V. The calculations were based on the following expression:^39^

$$C_{\mathrm{s}} = \frac{{{\int} {I\left( V \right){\mathrm{d}}V} }}{{2 \cdot s \cdot \Delta V \cdot A}}$$

where ${\int} {I\left( V \right){\mathrm{d}}V}$ is the total charge obtained by integration of the anodic and cathodic currents in a cyclic voltammogram, *s* is the scan rate, Δ*V* is the voltage range of the CV sweep, and *A* is the active surface area. The specific capacitances of the as-produced and the treated carbon electrodes at a 25 mV s^−1^ scan rate are 8.82 mF cm^−2^ and 46.64 mF cm^−2^, respectively, showing an increase of 5 times across various scan rates (Fig. 6e). The total improvement in specific capacitance can be credited to the improved electrode hydrophilicity as well as the increased hydroxyl, carbonyl, and carboxylic groups, which allow faster faradic reactions and increase the pseudocapacitance^12–15^. The results also indicate that plasma-treated electrodes exhibit a hybrid of pseudocapacitance and electrical double-layer capacitance properties.

The Faradaic electrochemical performance of carbon electrodes is often evaluated by using a ferri/ferrocyanide redox couple in solution^40–42^. In our experiments, a three-electrode electrochemical cell comprising a large carbon counter electrode, a Ag/AgCl reference, and a pyrolyzed carbon working electrode in a 1 mM K_3_ [FeCN_6_]/0.1 M KCL solution was used. The peak current and the peak-to-peak potential separation are important indicators of the charge transfer properties of an electrode surface. The anodic peak current density of the plasma-treated carbon electrodes is 790.51 µA cm^−2^ and that of the as-pyrolyzed carbon is 497.01 µA cm^−2^. The ratio of the anodic-to-cathodic peak currents (*I*_pa_/*I*_pc_) significantly improved from 0.55 in nontreated carbon to 0.98 after plasma direct writing. This improvement approaching 1 indicates a more reversible reaction on the electrode surface after treatment. The peak-to-peak separation (Δ*E*_*p*_) of the plasma direct-written surface was found to be 0.17 ± 0.02 mV compared with 0.5 ± 0.12 mV for the untreated electrode, also indicating faster electron transport and increased electrochemical reversibility after plasma treatment. This faster charge transfer with a functionalized carbon surface has been attributed to the presence of oxygen-containing groups, especially double-bonded C=O in carbonyl and carboxyl groups^14^. Our results show that microplasma direct-writing treatment enhances the electrochemical properties of carbon MEMS-manufactured electrodes by promoting charge transfer and thus making them more suitable for electrochemical sensor applications, e.g., in dopamine sensors. This surface treatment can also be used in the patterning of protein and DNA microarrays on C-MEMS electrodes because carboxyl-functional groups can be cross-linked with the terminal amines of biomolecules.

### Conclusions

We have demonstrated that microplasma direct writing assisted by water vapor is an efficient, rapid, and site-selective surface treatment technique. This targeted surface treatment can be used for the patterning of carbon electrodes with oxygen-functional groups at atmospheric pressure. We investigated the effect of both positive and negative corona plasma streamers on the functionalization efficiency. The negative plasma streamer treatment increased the atomic oxygen content from ~3 to 27% after 300 scan repetitions (180 s for a 4-mm length). The XPS findings indicate the enhancement of carbonyl, carboxylic, and hydroxyl groups for the treated carbon surface with carboxylic-functional groups showing the most improvement. Consequently, plasma direct writing improves the hydrophilicity and electrochemical characteristics of the carbon surface. The specific capacitance of the treated area is 46.64 mF cm^−2^ at a 25 mV s^−1^ scan rate, which shows a 5-fold increase from that of the nontreated carbon. Furthermore, microplasma-treated carbon surfaces also improve the electrochemical reversibility and yield faster electron transfer characteristics. Hence, this technique can be used to enhance bio- and electrochemical sensing performances, as well as the energy-storage efficiencies of carbon micro-/nanoelectrodes.

## Materials and methods

### Plasma direct-writing setup

A water vapor mist was generated by a vibrating mesh screen ultrasonic transducer that was in contact with a water-soaked sponge. The mesh with microsized holes was vibrated at ultrasonic frequencies, causing water on one side of the surface to be atomized and released as an aerosol mist from the other end. Then, the airflow (~500 sccm) serves as a carrier to bring the water vapor mist downstream to a nozzle. Sheath gas (either nitrogen or compressed air) was directed from a second outer nozzle, as shown in Fig. 1a. The sheath gas acts as a concentrating force for water vapor flow and removes deposited mini-water droplets on the electrode surface. The flow rates of the sheath and carrier gases were experimentally tuned to achieve optimum results. The nozzle housing was fabricated by 3D printing.

A tungsten wire electrode 100 μm in diameter was positioned at the center of the nozzle and connected to a high-voltage source. A radio-frequency voltage source of 5 kV at 5 MHz is applied between the tungsten wire tip and the conductive platform, which induces plasma discharge from the needle tip. Plasma discharge occurs when the needle tip is within a 4-mm distance from the target carbon electrode. The plasma current was stabilized by a high-impedance load connected in series with a voltage supply to prevent streams from propagating to arc discharge. The voltage source can be controlled from a computer over the timing and frequency of plasma through a microcontroller. The plasma stream ionizes water molecules generated from the back of the nozzle, creating reactive oxygen species that bombard the target. The nozzle was mounted on the 3-axis motion stage, which can be numerically controlled by g-code commands through the computer. The surface carboxylic functionalization degree was varied by exposure time over a target area. Direct writing of the functional groups is demonstrated by patterning on a 10-μm-thick SU-8-derived pyrolyzed carbon film fabricated by the carbon MEMS process. The details of the carbon structure fabrication by the C-MEMS process have been previously described in detail^41^.

### Surface characterization

After plasma direct writing, a nitrogen air gun was used to remove water residues on the carbon electrodes. Then, electrodes were dried in a drying oven at 60 °C for 2 h. In our analysis, FESEM units (Hitachi SU8030, Japan and Zeiss SIGMA, Germany) were used for image acquisition and EDS elemental measurement. C/O ratios were calculated from EDS and XPS measurements. FESEM images and EDS elemental maps were used to calculate the resolution of the patterning. XPS (PHI Quantera II, Chigasaki, Japan) was used to further verify the presence and percentage of carboxylic and other oxygen-functional groups on the electrode surface. The water contact angle was examined by a self-built apparatus comprising a horizontally mounted zooming camera and a droplet dispenser.

### Electrochemical measurements

The electrochemical responses from the plasma direct-written carbon surface and pristine carbon surface were investigated by using a µStat 400 multipotentiostat (DropSens-Metrohm, Asturias, Spain). A three-electrode configuration was used with a fabricated pyrolyzed carbon electrode as the working electrode, Ag/AgCl as the reference electrode, and a screen-printed carbon electrode as the counter electrode, and 0.5 M sulfuric acid was used as the electrolyte for evaluating specific capacitances and double-layer capacitances. A 1 mM K3 [FeCN6]/0.1 M KCL solution was used to evaluate the redox reaction. The solutions were deaerated by bubbling high-purity nitrogen gas for 15 min before measurements. For electrochemical measurements, area treatment of the electrodes was performed in a 3- × 4-mm area. Untreated electrodes have a 4- × 5-mm area in contact with solution. The current densities of the treated and untreated samples were normalized to compare the results in Fig. 6. The carbon electrode strips used in the experiments are illustrated in Fig. s3 of supplementary information.

## Supplementary information

Supplementary information
