# Supplementary material for: Microplasma direct writing for site-selective surface functionalization of carbon microelectrodes
Source: Microsyst Nanoeng. 2019 Nov 18;5:62. doi: 10.1038/s41378-019-0103-0 (PMC6859161; doi:10.1038/s41378-019-0103-0)
Supplement: Supplementary file 1 — Supplementary information [file 41378_2019_103_MOESM1_ESM.docx]

**Supplementary information**

# **Microplasma direct-writing for site-selective surface functionalization of carbon microelectrodes**

Aung Thiha^a,b^, Fatimah Ibrahim^a,b,*^, Shalini Muniandy^b,c^,Marc J. Madou^a,b,d,e^

^a^ Department of Biomedical Engineering, Faculty of Engineering, University of Malaya, 50603 Kuala Lumpur, Malaysia

^b^ Centre for Innovation in Medical Engineering (CIME), Department of Biomedical Engineering, Faculty of Engineering, University of Malaya, 50603 Kuala Lumpur,

Malaysia

^c^ Nanotechnology and Catalysis Research Centre, Institute of Graduate Studies, University of Malaya, 50603 Kuala Lumpur, Malaysia

^d^ Department of Biomedical Engineering, University of California, Irvine, CA 92697, USA

^e^ Department of Mechanical and Aerospace Engineering, University of California, Irvine, CA 92697, USA

## **XPS survey scan**

XPS survey scan of the untreated pyrolyzed carbon reveals mostly carbon peak at C1s and a small oxygen percentage can be observed in O1s peak. After plasma direct-writing, O1s improved significantly. Dissolved salts in water vapor source contributes to small peaks of sodium and calcium.

**Table S1**: Atomic concentrations as measured by XPS

-------------------------------------------------------------------------------------

C1s O1s Na1s Si2p S2p Ca2p

-------------------------------------------------------------------------------------

Untreated 95.55 3.90 - 0.54 - -

Treated 69.82 27.24 1.09 0.48 0.13 1.24


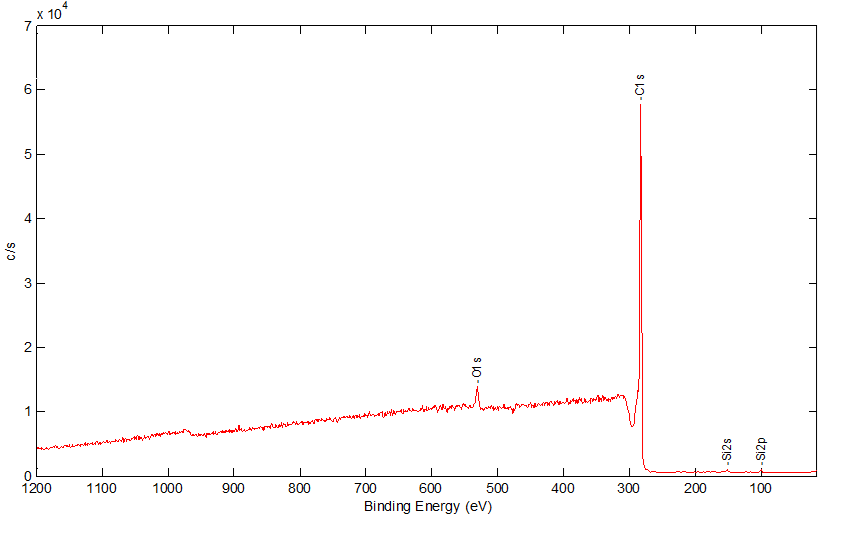

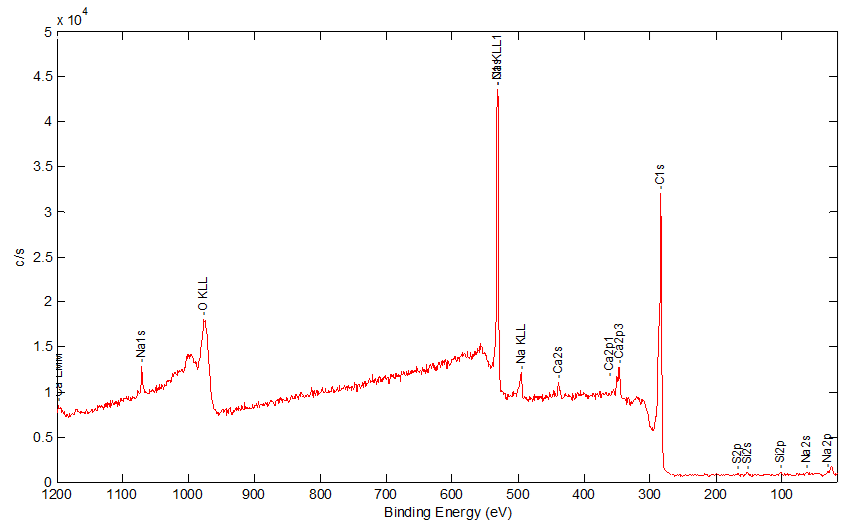


**a**

**b**

Fig.s1: **XPS survey scans. a** Untreated pyrolytic carbon. **b** Plasma direct-written pyrolytic carbon

## **Cyclic voltammograms (CV) at various scan rates**

**a**


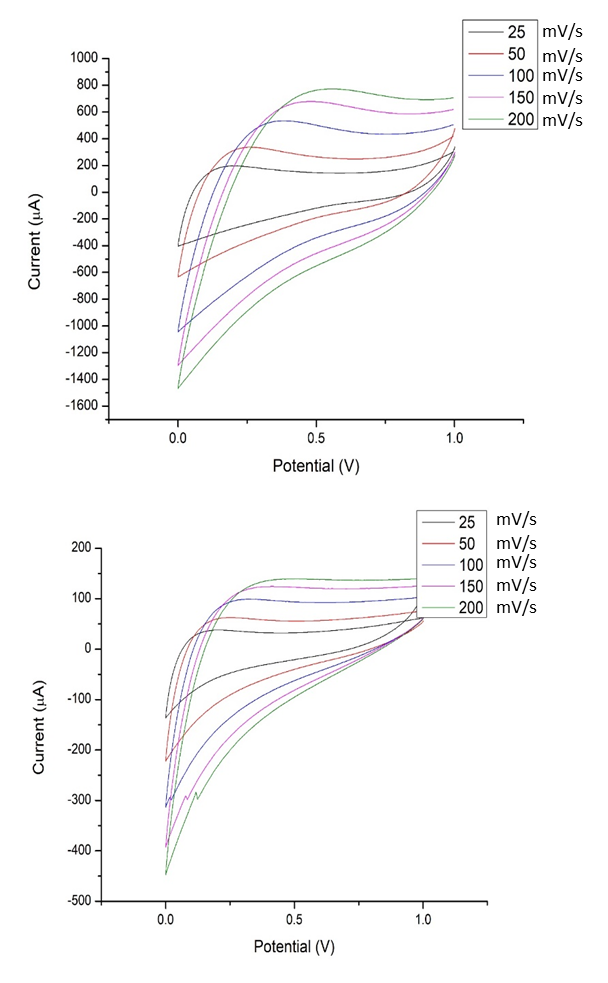


**Fig. s2: CVs** of (**a**) plasma direct-written surface and (**b**) untreated carbon surface at various scan rates.

**b**

The graphs show CVs performed in 0.5M H_2_SO_4_ at various scan rates using three-electrode electrochemical cells. The specific capacitances vs scan rates graph was constructed from the CV results.

## **Aging effect on functionalization**

An example of aging effect on functionalization is illustrated here. Patterned electrodes were stored in room conditions for 3 months before the second analysis at the same area.

1. Treated area 1


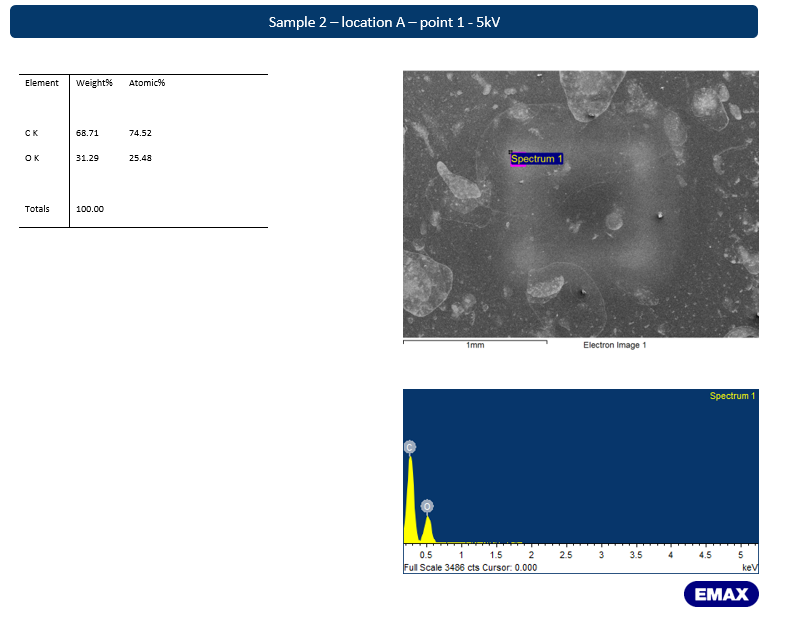


1. Treated area 1 after 3 months


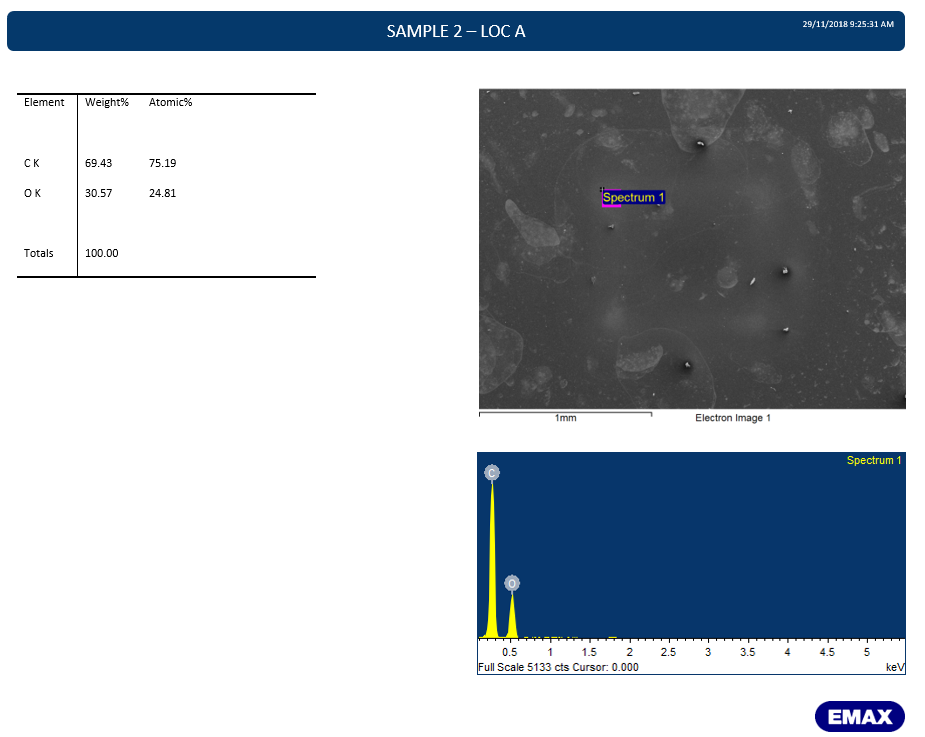


1. Untreated area


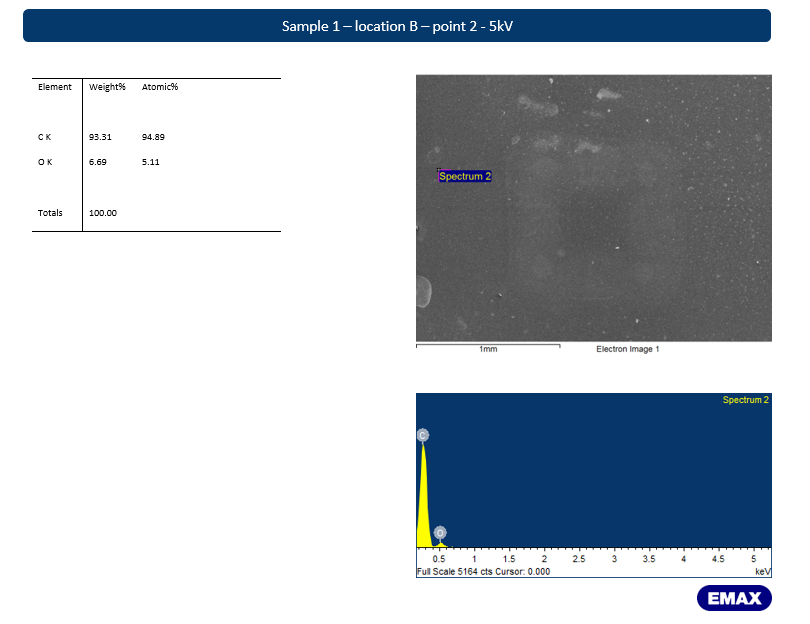


1. Untreated area after 3 months


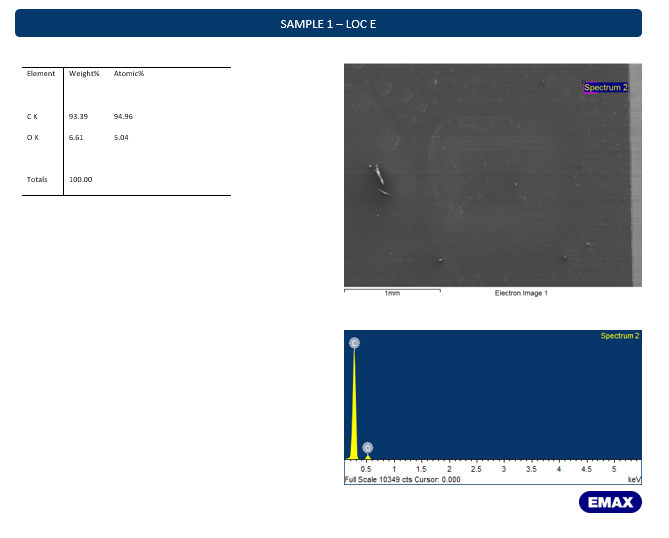


## **C/O Datasets**

The carbon to oxygen ratio achieved with various parameters are detailed in the following tables for reference.

**Variation in C/O and O % in various scanning repetitions.**

| In weight percent | | | | | | | | | | | | |
| --- | --- | --- | --- | --- | --- | --- | --- | --- | --- | --- | --- | --- |
| exposure | C/O weight ratio | | | | | | Oxygen weight percent | | | | | |
|  | Mean | Std dev | Max | min | p error | n error | Mean | Std dev | Max | min | p error | n error |
| 0 | 25.38 | 6.87 | 31.89 | 13.99 | 6.52 | 11.39 | 4.09 | 1.47433 | 6.67 | 3.04 | 2.58 | 1.05 |
| 20 | 17.85 | 3.30 | 21.22 | 14.63 | 3.38 | 3.22 | 5.42 | 0.95 | 6.40 | 4.50 | 0.98 | 0.92 |
| 50 | 10.19 | 1.40 | 11.97 | 8.56 | 1.78 | 1.63 | 9.04 | 1.13 | 10.46 | 7.71 | 1.42 | 1.33 |
| 100 | 6.81 | 0.47 | 7.15 | 6.48 | 0.34 | 0.34 | 12.82 | 0.78 | 13.37 | 12.27 | 0.55 | 0.55 |
| 150 | 4.58 | 0.73 | 5.17 | 3.76 | 0.59 | 0.82 | 18.15 | 2.52 | 21.00 | 16.21 | 2.85 | 1.94 |
| 200 | 3.91 | 0.40 | 4.41 | 3.30 | 0.49 | 0.61 | 20.46 | 1.74 | 23.24 | 18.50 | 2.78 | 1.96 |
| 250 | 2.57 | 0.27 | 2.76 | 2.38 | 0.19 | 0.19 | 28.10 | 2.15 | 29.62 | 26.58 | 1.52 | 1.52 |
| 300 | 2.06 | 0.29 | 2.26 | 1.73 | 0.20 | 0.33 | 32.89 | 3.30 | 36.68 | 30.70 | 3.79 | 2.19 |

| In Atomic Percent | | | | | | | | | | | | |
| --- | --- | --- | --- | --- | --- | --- | --- | --- | --- | --- | --- | --- |
| exposure | C/O atomic ratio | | | | | | Oxygen atom percent | | | | | |
|  | Mean | Std dev | Max | min | p error | n error | Mean | Std dev | Max | min | p error | n error |
| 0 | 34.91 | 10.70 | 48.13 | 18.65 | 13.21 | 16.27 | 3.05 | 1.19 | 5.09 | 2.03 | 2.04 | 1.02 |
| 20 | 23.77 | 4.38 | 28.24 | 19.49 | 4.47 | 4.28 | 4.12 | 0.73 | 4.88 | 3.42 | 0.76 | 0.70 |
| 50 | 13.58 | 1.87 | 15.95 | 11.41 | 2.37 | 2.17 | 6.95 | 0.89 | 8.06 | 5.90 | 1.12 | 1.05 |
| 100 | 9.08 | 0.63 | 9.53 | 8.63 | 0.45 | 0.45 | 9.94 | 0.62 | 10.38 | 9.50 | 0.44 | 0.44 |
| 150 | 6.10 | 0.97 | 6.89 | 5.01 | 0.79 | 1.09 | 14.28 | 2.08 | 16.63 | 12.68 | 2.35 | 1.60 |
| 200 | 5.21 | 0.53 | 5.87 | 4.40 | 0.65 | 0.81 | 16.19 | 1.46 | 18.52 | 14.56 | 2.33 | 1.63 |
| 250 | 3.42 | 0.36 | 3.68 | 3.16 | 0.26 | 0.26 | 22.69 | 1.87 | 24.01 | 21.37 | 1.32 | 1.32 |
| 300 | 2.75 | 0.40 | 3.04 | 2.30 | 0.28 | 0.45 | 26.85 | 3.01 | 30.30 | 24.78 | 3.45 | 2.07 |

**Variation in C/O and O % in various tungsten electrode tip to carbon surface distances**

| In Weight percent | |  |  |  |  |  |  |  |  |  |  |  |
| --- | --- | --- | --- | --- | --- | --- | --- | --- | --- | --- | --- | --- |
| Distance | **C/O weight ratio** | | | | | | **Oxygen weight percent** | | | | | |
|  | Mean C/O | Std dev | Max | min | p error | n error | Mean O2 % | Std dev | Max | min | p error | n error |
| 0.30 | 10.57 | 1.76 | 11.82 | 9.33 | 1.25 | 1.25 | 8.71 | 1.33 | 9.65 | 7.77 | 0.94 | 0.94 |
| 0.60 | 9.46 | 0.38 | 9.73 | 9.19 | 0.27 | 0.27 | 9.57 | 0.35 | 9.81 | 9.32 | 0.24 | 0.25 |
| 1.00 | 3.85 | 0.78 | 4.41 | 3.30 | 0.55 | 0.55 | 20.87 | 3.35 | 23.24 | 18.50 | 2.37 | 2.37 |
| 1.30 | 4.06 | 0.57 | 4.47 | 3.66 | 0.41 | 0.41 | 19.89 | 2.26 | 21.48 | 18.29 | 1.60 | 1.60 |
| 1.60 | 4.42 | 0.19 | 4.56 | 4.29 | 0.13 | 0.13 | 18.45 | 0.63 | 18.89 | 18.00 | 0.45 | 0.45 |

| Atomic percent | |  |  |  |  |  |  |  |  |  |  |  |
| --- | --- | --- | --- | --- | --- | --- | --- | --- | --- | --- | --- | --- |
| Distance | **C/O atomic ratio** | | | | | | **Oxygen atom percent** | | | | | |
|  | Mean C/O | Std dev | Max | min | p error | n error | Mean O2 % | Std dev | Max | min | p error | n error |
| 0.30 | 14.15 | 2.27 | 15.75 | 12.54 | 1.60 | 1.60 | 6.67 | 1.00 | 7.37 | 5.96 | 0.71 | 0.71 |
| 0.60 | 12.51 | 0.52 | 12.87 | 12.14 | 0.36 | 0.36 | 7.41 | 0.28 | 7.61 | 7.21 | 0.20 | 0.20 |
| 1.00 | 5.13 | 1.04 | 5.87 | 4.40 | 0.73 | 0.73 | 16.54 | 2.80 | 18.52 | 14.56 | 1.98 | 1.98 |
| 1.30 | 5.41 | 0.76 | 5.95 | 4.87 | 0.54 | 0.54 | 15.72 | 1.87 | 17.04 | 14.39 | 1.33 | 1.33 |
| 1.60 | 5.89 | 0.25 | 6.07 | 5.72 | 0.17 | 0.17 | 14.52 | 0.52 | 14.88 | 14.15 | 0.37 | 0.37 |

**Optical images of electrodes**


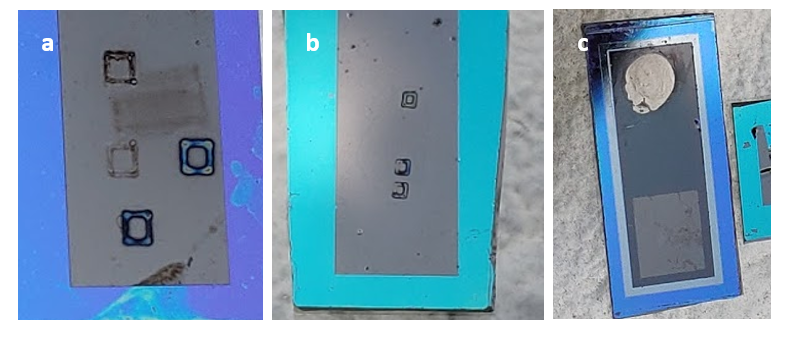


Fig. s3: Optical images of carbon film electrodes **a** 1 mm square patterns **b** 0.5 mm square pattern **c** Carbon film electrode used in electrochemical characterization. Square area is active electrode site. White circle is silver paste to connect with the electrochemical workstation.
